# Rapid Introgression of the Fusarium Wilt Resistance Gene into an Elite Cabbage Line through the Combined Application of a Microspore Culture, Genome Background Analysis, and Disease Resistance-Specific Marker Assisted Foreground Selection

**DOI:** 10.3389/fpls.2017.00354

**Published:** 2017-03-24

**Authors:** Xing Liu, Fengqing Han, Congcong Kong, Zhiyuan Fang, Limei Yang, Yangyong Zhang, Mu Zhuang, Yumei Liu, Zhansheng Li, Honghao Lv

**Affiliations:** Key Laboratory of Biology and Genetic Improvement of Horticultural Crops, Ministry of Agriculture, Institute of Vegetables and Flowers - Chinese Academy of Agricultural SciencesBeijing, China

**Keywords:** *Brassica oleracea* L. var. *capitata*, cabbage breeding, introgressive line, microspore culture, marker-assisted selection

## Abstract

Cabbage is an economically important vegetable worldwide. Cabbage Fusarium Wilt (CFW) is a destructive disease that results in considerable yield and quality losses in cole crops. The use of CFW-resistant varieties is the most effective strategy to mitigate the effects of CFW. 01-20 is an elite cabbage line with desirable traits and a high combining ability, but it is highly susceptible to CFW. To rapidly transfer a CFW resistance gene into 01-20 plants, we used microspore cultures to develop 230 doubled haploid (DH) lines from a cross between 01-20 (highly susceptible) and 96-100 (highly resistant). One of the generated DH lines (i.e., D134) was highly resistant to CFW and exhibited a phenotypic performance that was similar to that of line 01-20. Therefore, D134 was applied as the resistance donor parent. We generated 24 insertion–deletion markers using whole genome resequencing data for lines 01-20 and 96-100 to analyze the genomic backgrounds of backcross (BC) progenies. Based on the CFW resistance gene *FOC1*, a simple sequence repeat (SSR) marker (i.e., Frg13) was developed for foreground selections. We screened 240 BC_1_ individuals and 280 BC_2_ individuals with these markers and assessed their phenotypic performance. The proportion of recurrent parent genome (PRPG) of the best individuals in BC_1_ and BC_2_ were 95.8 and 99.1%. Finally, a best individual designated as YR01-20 was identified from 80 BC_2_F_1_ individuals, with homozygous *FOC1* allele and genomic background and phenotype almost the same as those of 01-20. Our results may provide a rapid and efficient way of improving elite lines through the combined application of microspore culture, whole-genome background analysis, and disease resistance-specific marker selection. Additionally, the cabbage lines developed in this study represent elite materials useful for the breeding of new CFW-resistant cabbage varieties.

## Introduction

Cabbage is an important vegetable that is cultivated worldwide. Cabbage Fusarium Wilt (CFW), which is caused by *Fusarium oxysporum* f. sp. *conglutinans* (Smith, 1899; Joseph, 1916), has recently become one of the most serious diseases threatening cabbage production in China. The occurrence and epidemic spread of this soil-borne disease is influenced by factors such as soil nutrient levels, temperature, and resistance of varieties (Steven et al., 2003). Appropriate soil temperatures (i.e., 22–28°C) are critical for the development of CFW infestations, and moisture (e.g., rain water or irrigation) accelerates the spread of this disease (Anderson, 1933; Li, 2004). The fungus responsible for CFW remains viable in soil for more than 10 years following its introduction. Additionally, CFW is difficult to control using physical or chemical methods.

According to the study of Lv et al. (2011), the inheritance of CFW resistance (Race 1) follows a monogenic dominant pattern, meaning CFW will be suppressed in F_1_ hybrids only if one of the parent lines is homozygous for the CFW resistance allele (Arden, 1979; Keinath et al., 1998; Farnham et al., 2001). Jones et al. (1920) were the first to identify several cabbage varieties resistant to CFW, including “Wisconsin All Seasons” and “Wisconsin Hollander.” Over the past few decades, CFW-resistant cabbage varieties have been continually bred and used commercially, such as “Badger Inbred-16” from the USA, “Rare Ball” from Japan, “Xiaqiang” from Korea, and “Zhonggan No. 18” from China.

Molecular-level studies on the genes associated with CFW resistance have recently laid the foundation for marker-assisted selection (MAS) in cabbage breeding programs. Pu et al. (2012) and Lv et al. (2013) have developed markers linked to the CFW resistance gene. Additionally, using doubled haploid (DH) and F_2_ cabbage populations, Lv et al. (2014a) fine-mapped a CFW resistance gene on an 84-kb interval, and identified a toll/interleukin-1 receptor-nucleotide biding site-leucine rich repeat (TIR–NBS–LRR) like gene (i.e., *FOC1*) as the candidate gene. Moreover, Shimizu et al. (2014a,b) determined that *FocBo1* may be responsible for CFW resistance in broccoli. These studies have facilitated other investigations into the molecular mechanisms regulating CFW resistance, and have accelerated the breeding of CFW-resistant cabbage varieties using MAS.

Gene transfers based on backcrossing are important for improving specific agronomic traits (Falcinelli, 1991; Vogel, 2009). However, this traditional breeding method depends on the selection of phenotypes, which are influenced by many factors, including environmental conditions as well as interactions between genes and between genotypes and environments (Ragagnin et al., 2003; Xu and Crouch, 2008). Additionally, this process usually takes seven or more years to develop an elite inbred line or variety. Marker-based background and foreground selections accelerate the elimination of undesirable background genetic material and transfer of desirable genes/traits, which considerably shortens the time required for breeding new varieties (Dwivedi et al., 2007; Tuberosa, 2013).

The culturing of isolated microspores has several advantages over anther culturing techniques, including the lack of regenerating anther tissue, and the fact it is less labor intensive and involves a shorter breeding cycle (Swanson et al., 1987). Lichter (1982) first reported the use of microspore cultures in *Brassica napus* experiments. Microspore cultures have since been used for many horticulturally important *Brassica oleracea* crops (Duijs et al., 1992).

We have used 01-20 as an elite cabbage line to breed more than 10 spring cabbage varieties (Lv et al., 2014b), including two excellent cabbage varieties (i.e., “8398” and “Zhonggan No. 21”) that account for over 70% of the spring cabbage market share in China. Especially “Zhonggan No. 21,” its cumulative harvesting areas has reached over 400,000 ha from 2006 to 2015. However, 01-20 is highly susceptible to CFW, which considerably limits its application. In this study, we used a microspore culture and MAS (i.e., genome-wide background markers and a resistance-specific foreground markers) to rapidly transfer a CFW resistance gene to 01-20 plants. Our data are relevant for future MAS studies, and this manuscript describes a faster way of improving elite breeding materials over currently used methods.

## Materials and methods

### Plant materials

**Figure 1:**
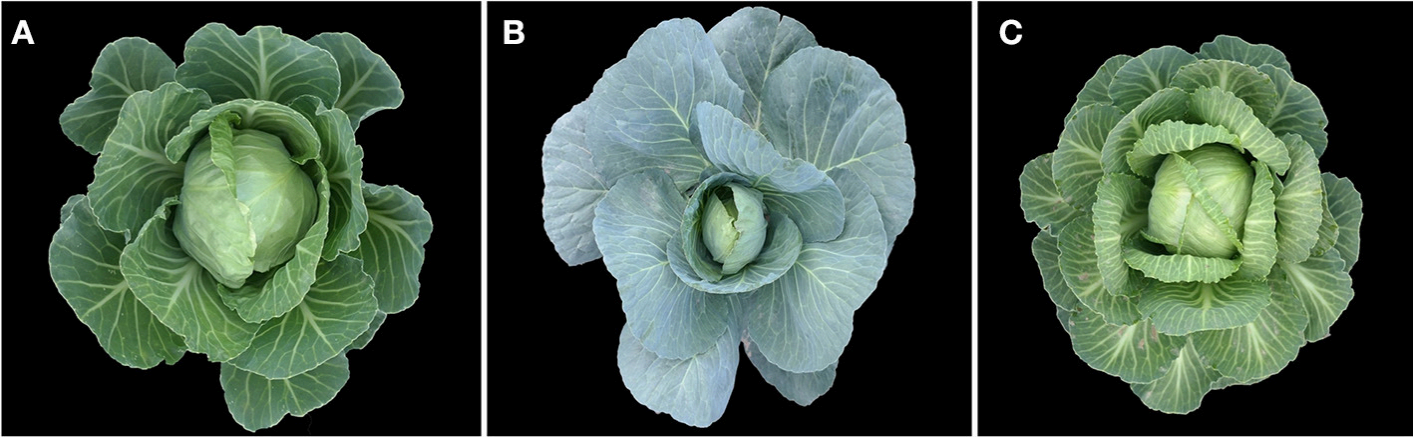
**Field performance of 01-20 (A)**, 96-100-308 **(B)**, D134 **(C)**.

The recurrent parent (i.e., line 01-20) was introduced to China from Canada in 1966 by the Institute of Vegetables and Flowers, Chinese Academy of Agricultural Sciences (IVF-CAAS). 01-20 is an early-maturing spring cabbage inbred line, with excellent agronomic characteristics and high combining ability (Figure 1A). The late-maturing autumn inbred line 96-100-308 (96-100 hereinafter) is highly resistant to Fusarium wilt (Figure 1B). We previously crossed lines 01-20 and 96-100, and generated 230 DH lines with isolated microspore cultures. Among them, 160 were generated between 2009 and 2011 (Lv et al., 2013), and another 70 lines were generated in 2011–2012. These lines were used to map the Fusarium wilt resistance gene (Lv et al., 2014a) and for quantitative trait locus analyses (Lv et al., 2016). Among these DH lines, D134 is highly resistant to Fusarium wilt in artificial inoculation assays, and is phenotypically similar to line 01-20 (Lv et al., 2014b; Figure 1C). Thus, D134 was selected as the source material for transferring the Fusarium wilt resistance gene into 01-20. Hybrid combination tests were performed in 2 consecutive years; 01-20 and D134 were crossed with another two lines 87-534 and CB201 separately; in both years hybrid combinations with D134 performed 20–30% lower yield than those with 01-20, which revealed that the combining ability of D134 was not as good as that of 01-20. Therefore, enhancing CFW resistance in cabbage line 01-20 is necessary. Additionally, 36 cabbage inbred lines with different resistance to Fusarium wilt (Table 1) were used to validate CFW resistance-specific markers. All plant materials were provided by the Cabbage and Broccoli Research Group, IVF-CAAS.

**Table 1:** **Geographic origins, maturity, head types, leaf colors, and resistances of 36 cabbage inbred lines**.

**Code**	**Name**	**Geographic origin**	**Maturity**	**Head type**	**Leaf color**	**Resistance to CFW**
1	QiuDe	Japan	Late	Oblate	Gray	HR
2	MingDe	Japan	Late	Oblate	Dark-green	HR
3	96-100	India	Early	Round	Gray-green	HR
4	Rare ball	Japan	Middle/Early	Round	Gray-green	HR
5	XiaQiang	Korea	Early	Round	Gray	HR
6	1038	Netherland	Middle/Early	Round	Dark-green	HR
7	YK-143	Japan	Late	Oblate	Gray-green	HR
8	AoQiNa	Japan	Late	Oblate	Gray-green	HR
9	HanTai	Japan	Middle/Late	Oblate	Dark-green	HR
10	YRHanDong	Japan	Middle/Late	Oblate	Dark-green	HR
11	HuaNai1	Japan	Middle/Late	Oblate	Dark-green	HR
12	YaFei	Japan	Middle/ Late	Round	Dark-green	HR
13	Russia05-17	Russia	Late	Round	Green	HR
14	HuBei1180	China	Early	Round	Green	HR
15	Kag91	Japan	Middle	Round	Gray-green	HR
16	308 DongSheng	Japan	Late	Oblate	Gray	R
17	02-12	China	Early	Pointed	Gray-green	HS
18	21-3	China	Middle	Oblate	Gray-green	HS
19	Tai41	Taiwan, China	Middle	Oblate	Dark-green	S
20	01-20	Canada	Early	Round	Green	HS
21	HeDaJiXin	Netherland	Early	Pointed	Dark-green	HS
22	PaTe	Netherland	Early	Round	Dark-green	S
23	1039	Netherland	Middle/Early	Round	Dark-green	HS
24	CB201	Thailand	Early	Round	Dark-green	HS
25	87-534	Germany	Early	Round	Green	HS
26	8282	Japan	Middle	Oblate	Yellow-green	HS
27	XiaJie	Japan	Middle	Oblate	Dark-green	HS
28	SiLiuSheng	Japan	Late	Oblate	Dark-green	HS
29	Russia05-13	Russia	Late	Round	Gray-green	HS
30	Minicole	Netherland	Early	Round	Gray-green	HS
31	QingGuang	Japan	Late	Oblate	Gray	S
32	LvQiu	Korea	Early	Round	Dark-green	HS
33	79-156	Denmark	Early	Round	Green	S
34	01-88	Canada	Early	Round	Green	S
35	JiBao	Japan	Middle	Round	Gray-green	S
36	Copenhagen	Denmark	Early	Round	Green	S

*HR, represent highly resistant; R, represent resistant; HS, represent highly susceptible; S, represent susceptible*.

### Development of primers for screening whole genome backgrounds

We designed 404 primer pairs for insertion–deletion (InDel) and simple sequence repeat (SSR) markers based on 96-100 and 01-20 resequencing data, using the *B. oleracea* reference genome sequence acquired from the *Brassica* database (http://brassicadb.org) was used as the “bridge” (Cheng et al., 2011). Each primer was designed with InDel or SSR variation of 3–5 bp, Tm-value of 54–56°C, GC content of 40–50%, and amplification product of 100–200 bp (Liu et al., 2013). These markers had been used for genetic mapping in our previous studies (Lv et al., 2013, 2016).

These 404 pair of primers was used for whole genome genomic background analyses in 01-20 and D134 to find the polymorphism markers on different chromosomal segments between them. According to the result of this analysis, parts of the polymorphic markers were chosen evenly distributed on the polymorphic region between D134 and 01-20 for genomic background analyses in backcross (BC) populations.

### Development of a cabbage fusarium wilt resistance-specific marker

Sequences upstream and downstream of *FOC1* were obtained from the 02-12 cabbage reference genome (http://brassicadb.org; Cheng et al., 2011; Lv et al., 2014a; Shimizu et al., 2014b) for detecting SSR and InDel loci and designing primers. Genomic DNA extracted from 96-100 and 01-20 plants was used to identify polymorphisms among the SSR markers. Additionally, 36 cabbage inbred lines with varied CFW resistance and 100 BC_1_ progeny plants identified by artificial inoculation were used to determine whether the Fusarium wilt resistance markers were versatile and stable.

The artificial inoculation of CFW was carried out for different cabbage materials, using the strain FGL3-6 of *F. oxysporum* f. sp. *conglutinans*. This strain was obtained from diseased plants in Yanqing County, Beijing, by the method of single spore isolation, and had been proven to be Race 1, with strong pathogenicity (Lv et al., 2011). The root-dipping method was used in artificial inoculation tests according to previous studies (Booth, 1971; Ramirez-Villupudua et al., 1985). We adjusted the conidia suspension to a concentration of 1 × 10^6^ conidia/ml using a hemacytometer, dipped the seedling (third leaf stage) root in the conidia suspension for 15 min, planted them in plastic pots (9 × 9 × 9 cm) with sterilized substrate (turf: vermiculite: soil = 1: 1: 2) and then cultivated in the greenhouse with a temperature of 25–28°C in the day and 18–22°C in the night. Ten days after inoculation we investigated the disease reaction.

### Molecular marker assay

Genomic DNA was extracted from fresh cabbage leaves using cetyltrimethylammonium bromide according to a slightly modified published method (Murray and Thompson, 1980). The genomic DNA concentrations were determined using the NanoDrop ND-100 spectrophotometer (Thermo Fisher Scientific Inc., Wilmington, DE, USA), and samples were then diluted to 40–50 ng/μL.

Polymerase chain reaction (PCR) experiments were conducted in 20-μL samples containing 4 μL DNA template (40–50 ng/μL), 2 μL 10 × PCR buffer (Mg^2+^ included), 1.6 μL dNTP (2.5 mM each), 0.8 μL forward and reverse primers (10 μM), 0.4 μL Taq DNA polymerase (2.5 U/μL), and 10.4 μL double-distilled H_2_O. The PCR was completed in a GeneAmp PCR system 9700 thermal cycler (Life Technologies Co., Carlsbad, CA, USA) using the following program: 94°C for 5 min; 35 cycles of 94°C for 30 s, 55°C for 30 s, and 72°C for 45 s; 72°C for 10 min. The amplified products were separated by 8% (w/v) polyacrylamide gel electrophoresis (160 V for 1.5 h). The amplicons were visualized with silver nitrate staining (Brant et al., 1991).

### Data collection and analysis

To identify the resistance genotype during foreground selections, the allele identical to that of the resistance-donor parent (i.e., D134) was recorded as “R” (i.e., resistant to CFW), while the allele identical to that of the recurrent parent (i.e., 01-20) was recorded as “S” (i.e., susceptible to CFW). For genetic background analyses, the alleles from D134 were recorded as “N,” while the alleles from 01-20 were recorded as “M.” The heterozygous alleles were categorized as “H.”

### Backcrossing and introgression of the CFW resistance gene into line 01-20

The donor parent D134 was crossed with the recipient parent 01-20 to obtain F_1_ generation plants, which were backcrossed with 01-20 to obtain the BC_1_ population. CFW resistance gene specific marker was used to identify the CFW resistance of all the BC_1_ individuals, and then according to the identification results, CFW-susceptible individuals were removed. All the CFW-resistant individuals were subjected to genomic background analyses using background makers. Individuals with most similar genomic background to 01-20 were selected for further BC process. These individuals were then backcrossed with 01-20 plants to produce the BC_2_ populations, which were subjected to the same screening process. And for calculating the Proportion of Recurrent Parent Genome (PRPG) more precisely, the best individuals in BC_1_ and BC_2_ were analyzed using all the 135 polymorphic markers between 01-20 and D134. The PRPG using the following formula: PRPG (%) = (L + X) /2L × 100%, where L represents the total number of valid markers, and X represents the number of markers identical to the recurrent parent (Hospital et al., 1992). Finally, individuals highly resistant to CFW with almost the same genetic background as 01-20 plants were self-pollinated to generate materials that were homozygous for the *FOC1* allele.

## Results

### Development of a simple sequence repeat marker closely linked to *FOC1*

**Figure 2:**
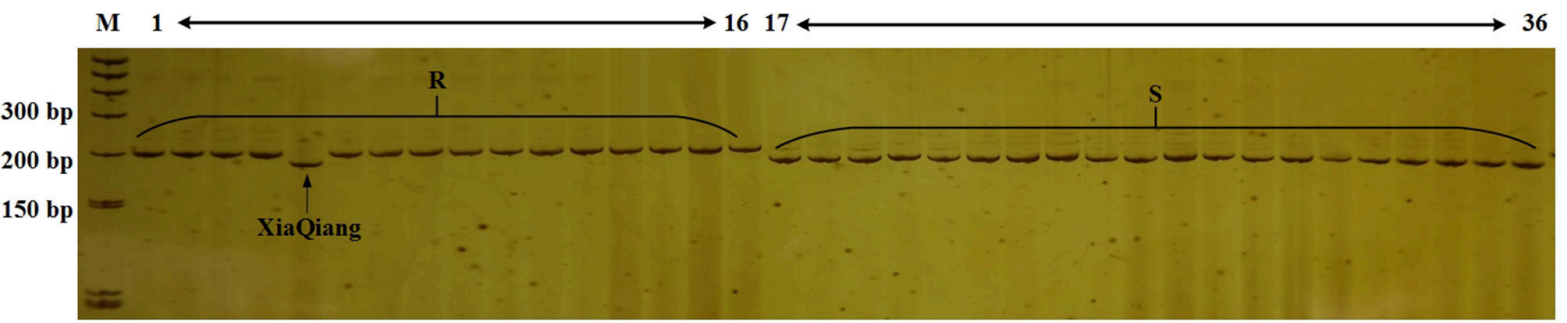
**Analysis of the Cabbage Fusarium wilt (CFW) resistance of 36 inbred lines using Frg13**. Lane M, size marker; Lanes 1–16, cabbage inbred lines resistant to CFW; Lanes 17–36, cabbage inbred lines susceptible to CFW.

Lv et al. (2013, 2014a) and Shimizu et al. (2014a,b) mapped the CFW resistance gene. However, we detected various mutant versions of the resistance gene in various cabbage materials. Additionally, molecular markers for identifying the resistance genotype exhibited a poor universality (data not shown). Therefore, we designed 66 primer pairs for SSR markers in the region spanning 100 kb upstream and downstream of *FOC1*. We also used previously developed markers in this region. Polymorphisms in these markers were detected in genomic DNA from lines 96-100 and 01-20. Eight polymorphic markers with distinguishable and reliable PCR products were used to analyze 36 inbred cabbage lines that exhibited different CFW resistance in artificial inoculation experiments. Among these SSR markers, Frg13 was located 75 kb from *FOC1*, between the marker Indel168 and Indel579 in C06 (**Figure 4**), and correctly detected the CFW resistance in 97.2% (35/36 × 100%) of the 36 inbred lines (Figure 2). “XiaQiang” was the only cabbage inbred line with inconsistent results between the molecular marker and artificial inoculation analyses. Thus, Frg13 may be useful for the molecular identification of CFW resistance in different cabbage materials and populations. The primer sequences for Frg13 are as follows: Forward primer (Frg13-F): 5′-ACCAGAGGCAGTTTTGGTTG-3′ (C06: 38827040…38827059); Reverse primer (Frg13-R): 5′-TCTTGCAACCCATGTCAAAA-3′ (C06: 38827176…38827195).

### Frg13-assisted identification of cabbage fusarium wilt resistance in backcross populations

**Figure 3:**
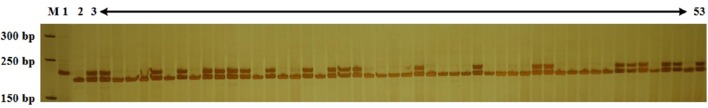
**Analysis of the Cabbage Fusarium wilt (CFW) resistance of 51 BC_2_ individuals using Frg13**. Lane M, size marker; Lane 1, resistance donor parent D134; Lane 2, recipient parent 01-20; Lane 3–53, BC_2_ individuals.

According to previous studies, cabbage resistance to Fusarium wilt race 1 is regulated by a single dominant gene (Walker, 1930; Walker and Hooker, 1945). Genomic DNA was extracted from all BC population individuals and analyzed with Frg13 to identify the CFW resistance genotype. D134 shared a large proportion of genetic background with 01-20. So a small BC population might be enough to obtain individuals with resistance to CFW and high PRPG. And actually, we screened 240 individuals for BC_1_ and 280 for BC_2_ populations. The results for some BC_2_ individuals are presented in Figure 3. We observed that 123 of 240 BC_1_ individuals and 134 of 280 BC_2_ individuals were resistant to CFW. The data of the foreground selection with Frg13 were presented in Supplementary Table 1. The segregation ratio for both BC_1_ and BC_2_ populations conformed to a Mendelian ratio of 1:1 according to a χ^2^-test. To validate the results of the marker analyses, we artificially inoculated 100 BC_1_ progeny plants by the root-dipping method according to previous studies (Booth, 1971; Ramirez-Villupudua et al., 1985). Among the inoculated 100 BC_1_ progeny plants we investigated 53 susceptible individuals and 47 resistant individuals, which was totally consistent with the results of the analysis using Frg13.

### Marker-assisted whole-genome background analysis

**Figure 4:**
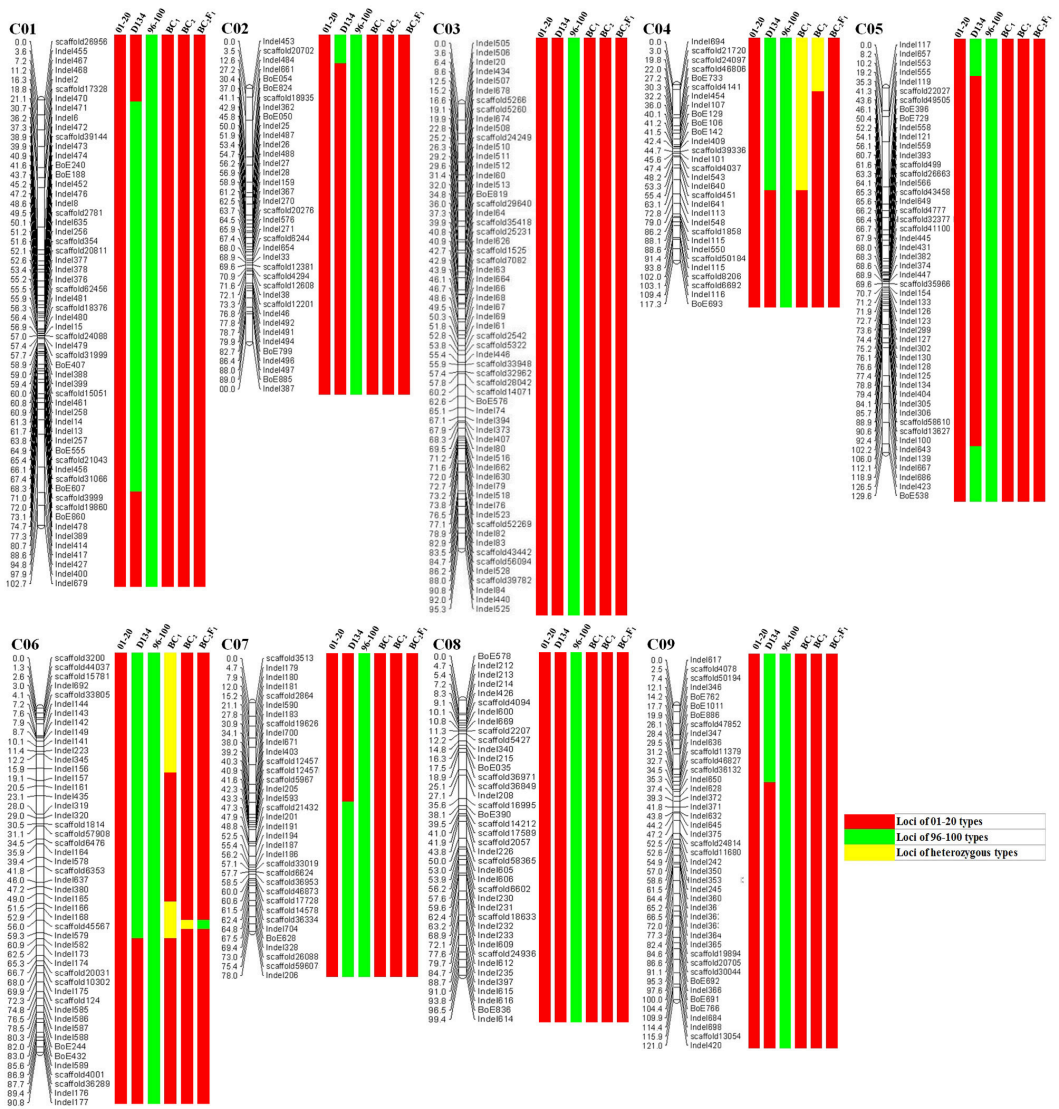
**Analysis of the genetic composition of 01-20, 96-100, D134, BC_1_, BC_2_, and BC_2_F_1_**. Marker locations and recombination distances (cM) are listed to the left; results of genetic background analysis are listed to the right of each marker locations. Red, Loci of 01-20 types; Green, Loci of 96-100 types; Yellow, Loci of heterozygous types.

The genetic structure of 01-20, 96-100, and D134 lines were analyzed with 404 previously described primer pairs (Figure 4). Hundred and thirty-five (indicated with green in Figure 4) among 404 loci showed polymorphism between 01-20 and D134; the remaining 269 loci (indicated with red in Figure 4) were identical between the two materials. We determined that 66.7% (269/404 × 100%) of the D134 genetic background was similar to that of line 01-20. The similarities were especially noticeable for chromosomes C03 and C08, in which the genetic composition was almost identical to that of line 01-20. Therefore, we focused on the 135 polymorphic loci in other seven chromosomes for marker selections. Finally, based on the genetic differences between lines D134 and 01-20, 24 primer pairs for polymorphic markers (indicated with blue in Supplementary Figure 1) were used to analyze the genetic backgrounds of 123 BC_1_ and 134 BC_2_ individuals with CFW resistance alleles. The 24 primer pairs evenly distributed on each polymorphic chromosome segment could be used as representatives of all the 135 polymorphic markers for the genetic background analysis. Primer details are provided in Table 2.

**Table 2:** **Details of markers used to screen genomic backgrounds**.

**Code**	**Marker**	**Marker type**	**Chromosome**	**Primer forward**	**Primer reverse**	**Product size**	**Tm (°C)**
1	InDel006	InDel	C01	TACCGAGTCACTCAGATTCC	GCTCTTATGGGCTTTGTTTA	170	55
2	InDel256	InDel	C01	GGAACTACCTGAATTCGACA	AAGGTGGAATCTACACATGG	156	53
3	InDel015	InDel	C01	ATAAGTTACGGCCAAGTCAA	GAATTTACAACGCCAACTTC	128	53
4	InDel257	InDel	C01	GGTTTCAGTCTTTGTACTTGC	CTGCAGTTACCATTCAGAGC	162	55
5	InDel453	InDel	C02	TGCACATGGCATAGTGATTA	ACACATGGTCTCCAACTCTC	112	52
6	InDel484	InDel	C02	CTGGAGACAGATGGTAAAGC	TGGTTTGTGTTCACAGGTAA	143	56
7	InDel694	InDel	C04	TTTTGCTGCCTAACAAGAGT	ACCTGAGAAGCTGCAGTAAT	189	55
8	InDel107	InDel	C04	TCTCCAAGGATTGTCATTTC	TGTGCCAGAAAGGAAACTAT	201	55
9	InDel409	InDel	C04	GCAGAACTGTTTCAGATCCA	CACCAGCGATAGAGTTTAGG	176	52
10	InDel543	InDel	C04	AACACGAACGGGTCTAGTAA	ATCCAATAGCTTGACCTTCA	143	53
11	InDel117	InDel	C05	CAGTTGAACTCGGGTCTTTA	TATGACAACTGCAAAAGTCG	120	53
12	InDel423	InDel	C05	GGCCGTGTATCTGTCATATT	CTAAAGCTGTACCTTGTGGC	135	55
13	InDel144	InDel	C06	GTGTTCATCGTTTTTAAGGC	ACTGGGTTGGGTTATTTATG	142	55
14	InDel142	InDel	C06	TGAGATGGAGAAAGAAAAGC	AAGTGTATCCCATTACCGTG	167	55
15	InDel320	InDel	C06	TGTTGTGTAGTGGAATCATCTC	TGATTAGACGGACCAACTTA	129	56
16	InDel579	InDel	C06	TGTTCCTGCTCCTCCTATAA	CAACGCTTTAGGTACTCCAT	146	56
17	InDel194	InDel	C07	ATAAGCGAAACAAGTGGAGA	AAATGTTCCTCATTCTGGTG	133	54
18	InDel186	InDel	C07	CAGGGTCGTAGAAGTGTCAT	AGACGCGCTTCTATTCTTAC	176	53
19	InDel328	InDel	C07	TATGTAAGAATCGTTTGGGC	GACCAGCTGTAACACGATTA	214	50
20	InDel206	InDel	C07	AATGACTACAATCAGGTGCC	CCAGACACTGAAGGAAAGAG	157	53
21	InDel617	InDel	C09	GTTGTACAGCTCTCAAAGGG	TAAAGAGGAGCTCCAACAAG	198	59
22	InDel346	InDel	C09	GGTTGAACTTTGGGTGAATA	GTACAACCTTGTCTGGGAAA	186	52
23	InDel347	InDel	C09	CTACGCCTTCCATTGTTTAC	GGGAGCGCTTTTAGAGTAAT	151	55
24	InDel636	InDel	C09	CAAAGGCTTCAGAAAAGTGA	GTAATATTCTTTGCAACCCG	188	54

Genetic backgrounds were investigated to identify the genomic constitution of the BC populations. The 123 BC_1_ individuals with CFW resistance alleles were screened using the 24 markers. We selected 12 individuals with genetic backgrounds similar to that of 01-20 plants for BCs with 01-20 to generate the BC_2_ plants whose backgrounds were also analyzed as described above. Finally, eight candidate BC_2_ individuals were obtained. The data of the background selection with 24 polymorphic markers were presented in Supplementary Table 1. Moreover, the best individuals in BC_1_ and BC_2_ were further analyzed using other polymorphic InDel and SSR markers. In this study, the total number of valid markers is 404, the best individuals in BC_1_ shared 370 identical markers with 01-20, and the best individuals in BC_2_ shared 397 identical markers with 01-20. So the *PRPG* was 95.8% for BC_1_ and 99.1% for BC_2_ (Figure 4).

**Figure 5:**
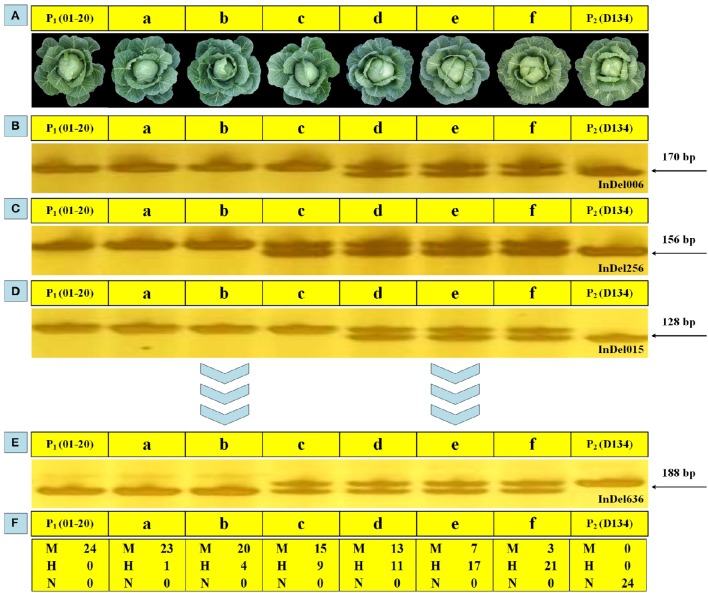
**Genomic backgrounds and phenotypic observations for six BC_1_ individuals. (A)** Phenotypic performance of 01-20, D134, and six BC_1_ individuals (a, b, c, d, e, and f) at the heading stage. **(B**–**E)** Polymerase chain reaction (PCR) amplification products for 01-20, D134, a, b, c, d, e, and f in using 24 whole-genome primer pairs. **(F)** Statistical analysis of the results for 24 primer pairs. M, the same allele as that of line 01-20; N, the same allele as that of line D134; H, heterozygous allele.

Analyzing the phenotype is a basic way to characterize the overall performance of various plant traits. In this study, except for using 24 markers to select genetic backgrounds during BCs, phenotypic observations were performed to screen BC populations to ensure appropriate individuals were selected. The plant expansion, leaf color, head shape, core length, and other parameters of BC_1_ and BC_2_ plants were examined, with D134 and 01-20 plants serving as reference materials. Six BC_1_ individuals (i.e., a–f) with different genetic background restoration rates were investigated, and the molecular characterization results were generally in accordance with the phenotypic observations. The individuals phenotypically more similar to 01-20 plants shared closer genetic background with 01-20 (i.e., identical markers; Figure 5). For the 24 loci genotyped by the 24 makers, individual “a” shared 23 loci with 01-20, while individual “f” shared only three loci with 01-20; so individual “a” was closer with 01-20 in genetic background, and phenotypically it was indeed more similar to 01-20, while individual “f” was similar to D134. Thus, phenotypic observations combined with maker assisted selection enabled a rapid and accurate selection of desirable plants from BC populations.

### Obtaining homozygous introgression lines

BC_2_F_1_ individuals were generated from self-pollinated progenies of the eight BC_2_ individuals. We identified the Fusarium wilt resistance of 80 BC_2_F_1_ individuals by the marker Frg13. There were 58 resistant individuals (17 with homozygous *FOC1* and 41 with heterozygous *FOC1*) and 22 susceptible individuals. The segregation ratio for BC_2_F_1_ populations conformed to a Mendelian ratio of 1:2:1. The genetic structure of the best BC_2_F_1_ individual was presented in Figure 4, except for the locus near *FOC1* on chromosome 6; the genetic background had been fully recovered. Additionally, this locus was previously confirmed to contain *FOC1* (Lv et al., 2014a). We designated the individual as YR01-20, and determined that it shared 99.8% (403/404 × 100%) genetic background with 01-20. Therefore, an investigation combining microspore cultures with MAS for disease resistance and whole-genome background analyses resulted in the generation of an introgression line that exhibits CFW resistance. This line is currently being applied for the breeding of new CFW-resistant cabbage hybrids.

## Discussion

*Fusarium oxysporum* f. sp. *conglutinans*, which is the pathogen responsible for CFW, can infect most *B. oleracea* crops, including cauliflower, broccoli, Brussels sprouts, kohlrabi, and Chinese kale (Kendrick, 1930; Pound and Fowler, 1951). The increasing occurrence and spread of CFW has necessitated the breeding of novel CFW-resistant varieties (Arden, 1979; Li et al., 2003; Zhang et al., 2008).

### Microspore cultures rapidly generate desirable cabbage lines

Doubled haploid lines are widely used in plant breeding programs because instead of requiring several generations of inbreeding through selfing, DH lines reach 100% homozygosity after just one generation *via* the induction of haploids. Thus, the development of new cultivars can occur relatively quickly (Jain et al., 1998; Castillo et al., 2001). The production of DH plants from microspores is an important technique used for plant breeding and basic research (Ferrie and Caswell, 2011). Isolated microspore cultures have been successfully used for *B. oleracea* crops, including cabbage (Rudolf et al., 1999), broccoli (Dias, 2001), and cauliflower (Stipic and Campion, 1997). Mejza et al. (1993) regenerated wheat plants from isolated microspores using an ovary co-culture. Although fruit species are considered to be recalcitrant to DH production, some progress has been made with apple (*Malus domestica* Borkh; Hofer, 2005). However, there are few reports describing the combined use of microspore cultures and BC breeding. In the current study, D134 is a DH line obtained through the culturing of isolated microspores. This line consists of a large proportion of the genomic background from the recurrent parent 01-20, which greatly improved the efficiency of backcrossing and decreased the number of generations required to produce a new line.

### Marker-assisted selection using whole-genome background markers and trait-specific markers is effective and efficient

Elite parental lines with a range of desirable agronomic properties and a high combining ability are considerably important for plant breeding programs (Zhuang, 2003). 01-20 is an elite parental line that has been useful for developing spring cabbage cultivars in China. One disadvantage of this line is that it is highly susceptible to CFW.

Backcross breeding is a traditional way to introduce a CFW resistance gene to 01-20 plants. During this procedure, which does not involve MAS, CFW resistance must be assessed for each individual through artificial inoculations. Several inoculation methods have been reported, including soaking roots in microspore suspensions and irrigation with inocula (Ramirez-Villupudua et al., 1985; Baik et al., 2010). This method is time-consuming and labor-intensive, and is easily affected by environmental conditions. Development of molecular markers closely linked to the CFW resistance gene may be a more efficient option. Jiang et al. (2011) developed an amplified fragment length polymorphism (AFLP) marker (transformed into sequence characterized amplified region marker S46M48199) closely linked to the CFW resistance gene at a genetic distance of 2.78 cM. Validations in an F_2_ population revealed that the results for the molecular marker were consistent with the artificial inoculation results in 81% of the cases. Pu et al. (2012) developed an SSR marker (i.e., KBrS003O1N10) associated with the CFW resistance gene at a genetic distance of 1.2 cM. Lv et al. (2013) developed InDel marker A1, which produced results that were in agreement with those of artificial inoculations in 96 and 82% of the F_2_ population and 40 inbred lines, respectively. Lv et al. (2014c) fine-mapped *FOC1* using InDel markers to a 84-kb region. We developed the Frg13 SSR marker closely linked to *FOC1* (0.1 cM) based on the candidate gene information and resequencing data for the two parents. This marker was validated in 100 BC_1_ individuals and 36 cabbage inbred lines, with results that were 100 and 97% identical to those of the artificial inoculations, respectively. Thus, Frg13 may be useful for accurately and rapidly identifying CFW resistance in these materials.

Another disadvantage of BC breeding is related to the undesirable chromosome fragments that may be introduced along with the target gene, which may adversely affect important agronomic traits (McCouch et al., 1988; Hittalmani et al., 2000). Markers for screening genomic backgrounds may help breeders to select individuals whose genetic background is most similar to that of the recurrent parent, thereby decreasing the number of required BC generations (Young and Tanksley, 1989). Naidoo et al. (2012) observed that when transferring *lpa1-1* to a maize inbred line, the restoration rate of the genetic background in BC_2_F_1_ plants reached 84% using marker-assisted screening of the genomic background. Similar findings were reported for rice regarding resistance to *Pyricularia oryzae* (84% in BC_3_; Gouda et al., 2013), tolerance to planthoppers, stem borers, leaf folders, and herbicides (95.8% in BC_3_F_4_; Wan et al., 2014), and the breeding of pepper varieties rich in capsaicin ester (96% in BC_2_F_1_; Jeong et al., 2015). In the current study, before the background analyses in background populations, genetic background of 01-20 and D134 were determined. The identical loci and chromosome fragments between 01-20 and D134 were supposed to be identical between BC individuals and 01-20. So to improve efficiency, only the polymorphic loci and chromosome fragments between 01-20 and D134 were analyzed in BC population. Thus, this study presents a rapid and effective way of generating introgressive lines through the combined application of microspore culture, backcrossing, genome-wide background analyses and resistance-specific marker assisted selection and phynotype evaluation.

## Conclusions

Combining microspore cultures with MAS (i.e., genomic background markers and CFW resistance-specific markers) resulted in the introgression of the CFW resistance gene to an elite cabbage inbred line. The cabbage lines YR01-20 developed in this study represent elite materials useful for the breeding of new CFW-resistant cabbage varieties. The CFW resistance-specific marker Frg13 will be useful for accurately and rapidly identifying CFW resistance in cabbage materials. Our data may be relevant for the development of an efficient method to improve elite cabbage lines.

## Author contributions

XL developed the markers performed the introgression of CFW resistance. XL, FH, and HL wrote and revised the manuscript. HL and ZF conceived the idea and reviewed the manuscript. CK, LY, YZ, MZ, YL, and ZL coordinated and designed the study. All the authors have read and approved the final manuscript.

## Funding

This work was financially supported by grants from the National Key Research and Development Program of China (2016YFD0100204), The Key Projects in the National Science and Technology Pillar Program during the Twelfth Five-Year Plan Period (2013BAD01B04-4), the Science and Technology Innovation Program of the Chinese Academy of Agricultural Sciences (CAAS-ASTIP-IVFCAAS), and the earmarked fund for the Modern Agro-Industry Technology Research System, China (nycytx-35-gw01).

### Conflict of interest statement

The authors declare that the research was conducted in the absence of any commercial or financial relationships that could be construed as a potential conflict of interest.

## Supplementary material

Supplementary Figure 1 **Distrubution of the 24 background polymorphic markers on each chromosome**.

Supplementary Table 1 **Information for the 24 background markers and one foreground marker used in BC_1_, BC_2_ and BC_2_F_1_**.
