# Supplementary figures and images for: Rapid Introgression of the Fusarium Wilt Resistance Gene into an Elite Cabbage Line through the Combined Application of a Microspore Culture, Genome Background Analysis, and Disease Resistance-Specific Marker Assisted Foreground Selection
Source: Front Plant Sci. 2017 Mar 24;8:354. doi: 10.3389/fpls.2017.00354 (PMC5364174; doi:10.3389/fpls.2017.00354)

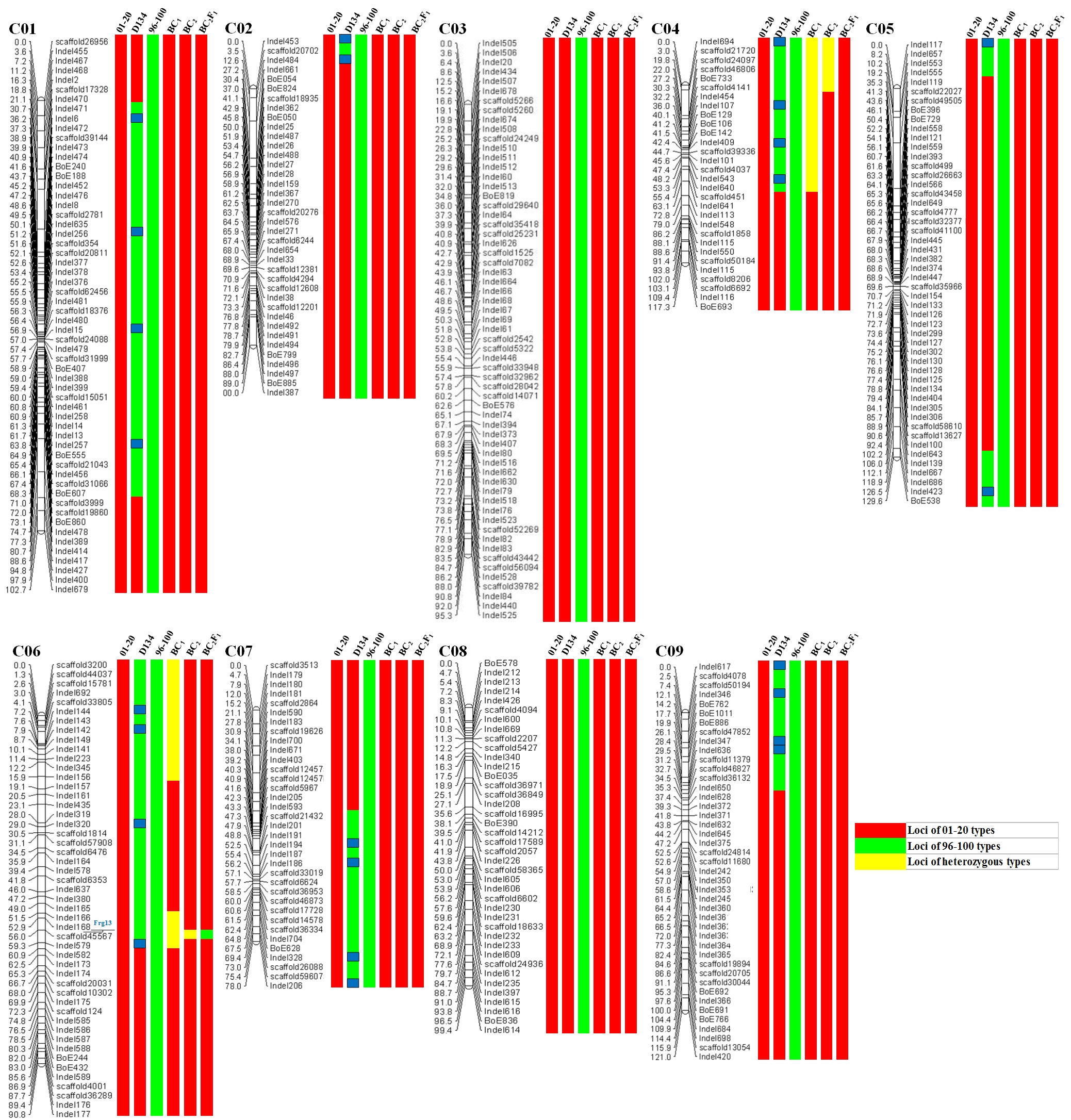

Supplement: Supplementary Figure 1 — Distrubution of the 24 background polymorphic markers on each chromosome. [file Image1.JPEG]
